# Hypoxia and HIF signalling in tumour microenvironment: linking immune evasion, metabolic rewiring and epigenetic regulation

**DOI:** 10.1017/erm.2026.10044

**Published:** 2026-03-27

**Authors:** Shadiya Fawzul Ameer, Elham Abdul Latif M Sharif, Wisam Nabeel Ibrahim

**Affiliations:** Department of Biomedical Sciences, College of Health Sciences, QU Health, Qatar University, Doha, Qatar

**Keywords:** HIF, hypoxia, immune resistance, metabolic reprogramming, TME

## Abstract

**Background:**

Hypoxia is a defining feature of the tumour microenvironment (TME) that drives aggressive tumour behaviour through coordinated adaptive responses. Hypoxia-inducible factors (HIFs), particularly HIF-1α, play a central role in orchestrating metabolic, immune and epigenetic reprogramming within tumours.

**Objective:**

This review aims to elucidate the integrated roles of hypoxia in regulating angiogenesis, immune suppression, metabolic adaptation and epigenetic modifications, and to highlight their collective impact on tumour progression and therapeutic resistance.

**Methods:**

A comprehensive review of current literature was conducted to examine the molecular and cellular mechanisms mediated by hypoxia and HIF signalling within the TME, with a focus on their interplay across angiogenic, immune, metabolic and epigenetic pathways.

**Results:**

HIF-1α promotes the expression of pro-angiogenic factors, including VEGF, ANGPT2 and CXCL12, leading to abnormal vascularisation and recruitment of immunosuppressive cells such as regulatory T cells and myeloid-derived suppressor cells. This disorganised vasculature exacerbates hypoxia, reinforcing a cycle of immune evasion and metabolic stress. Hypoxia also upregulates immune checkpoint molecules (e.g., PD-L1, PD-1), contributing to T-cell exhaustion and impaired dendritic cell function. Concurrently, metabolic reprogramming—characterised by increased glycolysis, lactate accumulation and extracellular acidification—suppresses cytotoxic T cell and NK cell activity. Epigenetic regulators, including histone demethylases and DNA methyltransferases, sustain these adaptations through persistent transcriptional changes, referred to as hypoxic memory.

**Conclusion:**

Hypoxia acts as a central organising force within the TME, coordinating angiogenic, immune, metabolic and epigenetic processes to promote tumour progression. Targeting HIF-driven pathways represents a promising therapeutic strategy to overcome immune resistance, enhance drug delivery and improve the efficacy of combination treatments, including immunotherapy and metabolic interventions. This review underscores the importance of integrated approaches to disrupt hypoxia-mediated tumour adaptation.

## Introduction

Cancer is not merely a disease of unchecked cell division but a complex, adaptive process that is profoundly influenced by the tumour microenvironment (TME) – a heterogeneous network of immune cells, stromal components, vasculature and extracellular matrix (Ref. 1). One of the most defining and deleterious features of the TME is hypoxia, a condition of oxygen deprivation that arises as rapidly proliferating tumour cells outstrip their blood supply (Ref. 2). Far from being a passive consequence, hypoxia actively shapes tumour evolution and has been strongly associated with poor prognosis, metastasis and resistance to therapy in multiple cancer types (Ref. 3).

Clinically, hypoxia and its mediators, especially hypoxia-inducible factors (HIFs), are appealing therapeutic targets. By inhibiting HIF activity or its downstream effectors, it may be possible to reverse immune suppression, improve immune cell infiltration and make tumours more responsive to immunotherapies and chemotherapies. Moreover, therapies that correct hypoxia-induced abnormal blood vessel growth or mitigate hypoxia-driven metabolic alterations could enhance drug delivery and decrease tumour aggressiveness. These strategies are currently being explored in preclinical and clinical studies, underscoring the importance of comprehending hypoxia’s impact on tumour biology (Refs. 4, 5).

The central mediators of the hypoxic response are HIFs, transcriptional regulators that orchestrate a wide array of genetic programmes enabling tumour cells and stromal elements to adapt and thrive under low oxygen conditions (Ref. 6). HIF-1α induces the expression of genes involved in angiogenesis (e.g., VEGF, ANGPT2, CXCL12), metabolic reprogramming (e.g., GLUT1, lactate dehydrogenase A [LDHA]) and immune suppression (e.g., PD-L1, CCL28), fostering a microenvironment conducive to immune evasion and tumour expansion (Ref. 7).

Under hypoxic conditions, the tumour immune landscape is profoundly altered. There is increased recruitment of immunosuppressive cell populations, including regulatory T cells (Tregs), myeloid-derived suppressor cells (MDSCs) and M2-polarised macrophages. These cells contribute to the suppression of cytotoxic CD8+ T lymphocytes, promote immune checkpoint expression (e.g., PD-1/PD-L1) and blunt antigen presentation by dendritic cells, ultimately enabling tumours to escape immune surveillance (Ref. 8). Compounding this, hypoxia-induced angiogenesis not only fuels tumour growth but also impedes effective immune cell infiltration and function, further promoting an immunosuppressive milieu (Ref. 9).

In parallel, metabolic adaptations such as the shift from oxidative phosphorylation to aerobic glycolysis – the Warburg effect – create a hostile environment for effector immune cells due to acidosis and competition for nutrients. Furthermore, hypoxia and HIF signalling drive epigenetic alterations via histone modifications and DNA methylation, locking in gene expression profiles that favour tumour cell survival, stemness and immune resistance (Ref. 10).

This review explores the multifaceted impact of hypoxia on tumour biology, focusing on how it orchestrates a vicious cycle of immune suppression, metabolic rewiring, angiogenesis and epigenetic plasticity. By dissecting these interlinked pathways, we aim to clarify the central role of hypoxia in tumour progression and highlight key molecular events that may serve as targets for disrupting this adaptive network.

## Hypoxia in the tumour microenvironment

The TME is a highly structured and dynamic ecosystem where cancer cells interact with a variety of non-malignant cells, all embedded within a remodelled and vascularised extracellular matrix (ECM). This environment comprises immune cells, cancer-associated fibroblasts, endothelial cells, adipocytes and pericytes, collectively driving molecular, cellular and physical changes that facilitate cancer progression, metastasis and therapeutic resistance (Refs. 1, 11–14).

One of the most defining characteristics of the TME is hypoxia, particularly in solid tumours, where rapid cell proliferation and high metabolic demand result in an uneven oxygen distribution (Refs. 2, 5). Hypoxia, which affects up to 90% of solid tumours, is a key hallmark of cancer and is associated with poor prognosis in malignancies, such as prostate, cervical and head and neck squamous cell carcinoma. Beyond promoting cancer progression, hypoxia enhances tumour cell proliferation, alters gene expression and drives metabolic adaptation (Refs. 15–17).

The hypoxic response is primarily regulated by HIFs, transcription factors that orchestrate gene expression in response to oxygen deprivation. HIFs are heterodimers composed of an oxygen-sensitive alpha (HIF-α) subunit and a constitutively expressed beta (HIF-β) subunit. Among these, HIF-1α is the most well-characterised and plays a pivotal role in mediating hypoxic adaptation in mammalian cells (Ref. 18).

Under normoxic conditions (~8% O2, 60 mmHg), HIF-1α undergoes hydroxylation at proline residues P402 and P564 by prolyl hydroxylase domain proteins (PHD1–3) (Ref. 19). This hydroxylation facilitates recognition by the von Hippel–Lindau tumour suppressor protein (pVHL), which recruits an E3 ubiquitin ligase complex to ubiquitinate HIF-1α, leading to its rapid proteasomal degradation. As a result, HIF-1α is constantly synthesised and degraded under normal oxygen levels, preventing unnecessary hypoxic signalling. However, in hypoxic conditions, PHD activity is inhibited, allowing HIF-1α to stabilise, translocate to the nucleus, dimerise with HIF-1β and recruit transcriptional coactivators such as p300/CBP. This complex binds to hypoxia response elements (HREs) within gene promoter regions, activating downstream pathways that enhance tumour aggressiveness, metastasis and resistance to treatment (Refs. 2, 3, 7).

HIF-1α also modulates cancer metabolism by promoting glycolysis, increasing the expression of glycolytic enzymes and glucose transporters, thereby enabling cancer cells to thrive under oxygen-deprived conditions. While HIF-1α regulates many hypoxia-responsive genes, HIF-2α has a more cell-type-specific role. Also known as endothelial PAS domain protein 1 (EPAS1), HIF-2α stabilises under hypoxia and interacts with the aryl hydrocarbon receptor nuclear translocator (ARNT). Unlike HIF-1α, HIF-2α remains active under moderate hypoxia (<5% O_2_) and persists for longer periods (48–72 hours), suggesting a functional distinction in which HIF-1α responds to acute hypoxia while HIF-2α governs chronic hypoxic adaptation (Refs. 20, 21).

HIF-3α, though less studied, appears functionally distinct from its counterparts. Unlike HIF-1α and HIF-2α, which contain two transactivation domains (TADs), HIF-3α has only one, along with a unique leucine zipper domain and an LXXLL motif. Some splice variants, such as the inhibitory PAS domain protein (IPAS), act as dominant-negative inhibitors of HIF-1α. Despite its relatively low sequence similarity with HIF-1α and HIF-2α, the evolutionary conservation of HIF-3α suggests a specialised regulatory role in hypoxia adaptation (Refs. 15, 22).

Although HIF-1α and HIF-2α share 48% amino acid sequence identity, their functional roles are distinct: HIF-1α primarily mediates acute hypoxia responses, whereas HIF-2α is crucial for chronic hypoxic adaptation. This regulatory shift enables tumour cells to sustain oxygen-deprived conditions over time. Interestingly, HIF-1α and HIF-2α can exert opposing but complementary effects, highlighting the intricate balance that tumour cells employ to navigate hypoxia (Ref. 6).

Beyond its role in hypoxia adaptation, HIF-1α has been extensively implicated in tumour growth and angiogenesis (Ref. 23). Gene deletion studies in mouse embryonic stem cells revealed that the absence of HIF-1α reduces tumour mass and enhances apoptosis in teratocarcinomas (Ref. 24). Similarly, subcutaneous xenograft mouse models have demonstrated that HIF-1 promotes tumour vascularisation and expansion (Ref. 21). In human studies, HIF-1α has been positively correlated with vascular endothelial growth factor (VEGF) expression in brain and breast cancers, further underscoring its role in angiogenesis. Moreover, transgenic cancer models have established HIF-1α as a key driver of tumour progression and metastasis (Ref. 22).

The molecular regulation of HIF-1α is complex, involving multiple levels of control over its expression, stability and activity. Under normoxic conditions, HIF-1α is primarily regulated at the level of protein stability, where oxygen-dependent hydroxylation by PHD enzymes promotes pVHL-mediated ubiquitination and degradation (Refs. 25, 26). Additionally, factor-inhibiting HIF-1 (FIH) hydroxylates an asparagine residue in HIF-1α’s C-terminal TAD (TAD-C), preventing coactivator binding and limiting transcriptional activity. Furthermore, activation of the p42/p44 MAPK pathway enhances HIF-1α transcriptional activity by promoting nuclear accumulation, a process independent of oxygen levels (Ref. 6).

HIF-1α expression is also regulated at the mRNA level through the PI3K/AKT signalling pathway. Growth factors and receptor tyrosine kinase mutations that disrupt tumour suppressor genes or activate oncogenes can enhance PI3K pathway signalling, leading to increased tumour growth. Activation of PI3K/AKT signalling enhances HIF-1α activity, while loss of PTEN, a tumour suppressor that inhibits AKT activation, further amplifies HIF-1α levels. Additionally, insulin-like growth factor 1 (IGF-1) has been shown to induce HIF-1α activity via PI3K/AKT signalling (Ref. 27).

Overall, hypoxia-induced HIF activation plays a central role in metabolic reprogramming, angiogenesis and tumour adaptation, ultimately driving cancer progression and therapy resistance.

## The role of cancer-associated fibroblasts in hypoxic tumour niches

Hypoxia significantly influences not only malignant epithelial cells but also stromal components, such as cancer-associated fibroblasts (CAFs). Oxygen deprivation changes CAF behaviour and function through mechanisms involving both HIF-dependent and HIF-independent pathways. These changes affect the ECM, immune responses, metabolic processes, blood vessel formation, metastasis and resistance to therapy (Ref. 28). At the molecular level, the HIF family, especially HIF-1α and HIF-2α, plays a central role. Their stability is regulated by prolyl hydroxylase domain enzymes (PHD/EGLN) and factor inhibiting HIF (FIH) (Ref. 29). Under normal oxygen conditions, HIF-α is hydroxylated on proline residues, recognised by the von Hippel–Lindau (VHL) E3 ligase complex, ubiquitylated and degraded (Ref. 30). During hypoxia, PHD activity decreases due to lower oxygen levels and less α-ketoglutarate-dependent hydroxylation, leading to stabilisation of HIF-α. This allows HIF-α to form a heterodimer with ARNT (HIF-β), attract CBP/p300 coactivators and activate transcription of genes with HREs (Ref. 31). Notably, CAFs respond strongly to HIF activation by integrating hypoxic signals with TGF-β, PI3K/AKT, ERK and ROS signalling pathways (Ref. 32).

Hypoxia in CAFs induces ECM remodelling by activating collagen-modifying enzymes such as P4HA1/2 and PLOD2, which hydroxylate proline and lysine residues in procollagen, thereby stabilising the triple helix and promoting crosslinking. Lysyl oxidase-like 2 (LOXL2) further promotes collagen fibre cross-linking, increasing ECM stiffness and tensile strength (Ref. 33). This rigidity enhances integrin signalling, mechanotransduction and tumour cell invasion. Hypoxia also elevates TIMP1, thereby affecting ECM synthesis and degradation, and upregulates enzymes, such as MMP2, MMP9, MMP14, cathepsin D and uPAR. As a result, hypoxic CAFs regulate both matrix production and degradation, forming aligned collagen tracks that facilitate cancer cell invasion and intravasation (Ref. 34). Additionally, HIF-1–driven CA9 expression acidifies the extracellular environment, activating MMPs and degrading the matrix, while protecting cells from pH stress inside the cells (Ref. 28).

Hypoxia also influences CAF-driven immune regulation. HIF-1α increases TGF-β production, encouraging fibroblasts to become myofibroblasts, characterised by α-SMA in stress fibres. These CAFs release CXCL12 (SDF-1), which interacts with CXCR4 on tumour and immune cells. CXCR4 is a HIF-1α target gene, and hypoxia stabilizes its mRNA, enhancing its expression (Ref. 35). This chemokine pathway supports tumour cell migration and blood vessel formation and prevents cytotoxic T cell infiltration. Hypoxic CAFs additionally produce IL-6, IL-10, PD-L1 and ARG2. The expression of ARG2, driven by HIF-1α, reduces extracellular arginine levels, disrupting T-cell receptor signalling, proliferation and effector functions (Ref. 36). Consequently, hypoxic CAFs create a metabolically restrictive and immunosuppressive microenvironment. Radiation therapy may exacerbate this condition by generating reactive oxygen species and altering oxygen levels, thereby further activating fibroblasts and increasing fibrotic and pro-angiogenic responses.

Metabolic reprogramming is another key feature of hypoxic CAF activity. Stabilised HIF-1α promotes glycolytic enzymes like PDK1 and PKM2, enhances GLUT1 presence on the cell membrane and boosts lactate production (Ref. 37). ROS-activated ATM kinase phosphorylates GLUT1 and increases PKM2 levels, driving aerobic glycolysis in CAFs (Ref. 38). Lactate exported via MCT4 is taken up by tumour cells, fuelling anabolic processes such as the pentose phosphate pathway and nucleotide synthesis (Ref. 39). In prostate cancer, SIRT3-dependent ROS stabilizes HIF-1α in CAFs, increasing GLUT1 and MCT4 expression and facilitating lactate transfer (Ref. 40). Moreover, reduced IDH3α in CAFs lowers α-ketoglutarate levels, impairing PHD2 activity and stabilising HIF-1α independently of oxygen availability (Ref. 41). This α-KG–dependent pathway maintains HIF signalling, shifts metabolism towards glycolysis and inhibits oxidative phosphorylation by inducing NDUFA4L2. Hypoxic CAFs also trigger BNIP3-mediated autophagy, leading to Caveolin-1 degradation and, in turn, further HIF-1α stabilisation via ROS feedback (Ref. 42). Autophagy supports nutrient recycling and promotes exosome release via ATM-mediated phosphorylation of ATP6V1G1, aiding tumour-promoting paracrine signalling.

In angiogenesis, hypoxic CAFs increase VEGF production via an HIF-1α-dependent pathway and often interact with oestrogen receptor signalling, including GPER (Ref. 43). The VEGF produced by CAFs activates endothelial VEGFR, promoting cell proliferation and sprouting. Interestingly, deleting HIF-1α specifically in fibroblasts unexpectedly normalizes tumour blood vessels and improves blood flow, suggesting that excessive HIF-driven angiogenesis leads to abnormal, dysfunctional vessels. Proteomic analyses reveal that hypoxic CAFs also secrete additional pro-angiogenic factors, such as STC1 and HIAR, which further influence endothelial behaviour (Ref. 44). Therapeutically, combining anti-VEGF drugs, such as bevacizumab, with immune checkpoint inhibitors improves treatment outcomes, underscoring the link between angiogenesis and immune response (Ref. 45).

Metastatic progression is closely connected to hypoxic CAF signalling. Altering PHD2 influences CAF activation and ECM contraction. PHD2 deficiency can impair TGF-β1-driven paracrine communication between cancer cells and CAFs, thereby affecting matrix formation and spread (Ref. 46). In some cases, stabilising HIF-1α in CAFs lowers α-SMA and periostin levels, reducing pro-metastatic ECM remodelling (Refs. 47, 48). On the other hand, hypoxic CAFs release hepatocyte growth factor (HGF), which activates c-Met in pancreatic cancer cells, and produce ROS that activate the NF-κB and COX-2 pathways, thereby supporting epithelial–mesenchymal transition (EMT) (Ref. 49). Additionally, hypoxia-induced exosome secretion transfers signalling molecules that reprogram cancer cell transcription, further promoting metastasis.

Therapeutically, targeting pathways such as TGF-β, HIF, CXCL12/CXCR4 and integrin αvβ3 has shown promise. Drugs such as tranilast and pirfenidone block TGF-β- driven activation of CAFs, while minnelide inhibits both TGF-β and HIF-1 transcriptional activities (Ref. 50). Belzutifan, which targets HIF-2α, demonstrates the clinical application of hypoxia-focused therapy (Ref. 51). CXCR4 antagonists, such as AMD3100, interfere with CAF-mediated immune exclusion (Ref. 52). Nonetheless, CAF heterogeneity presents challenges: removing α-SMA-positive CAFs may increase tumour hypoxia and immune checkpoint expression, potentially accelerating tumour growth.

**Figure 1.**
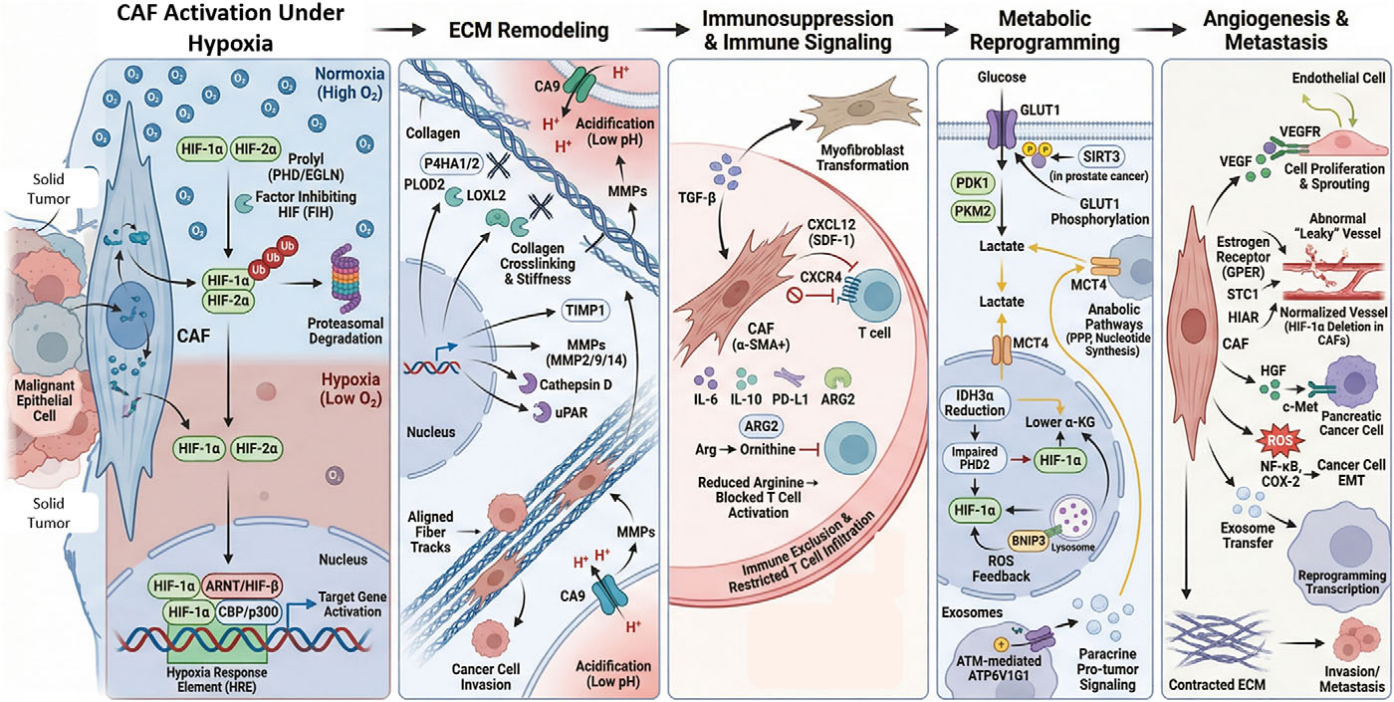
Hypoxia-driven activation of cancer-associated fibroblasts (CAFs) promotes tumour progression through ECM remodelling, immune suppression, metabolic reprogramming and metastasis. Under hypoxia, HIF-1α/HIF-2α stabilize in CAFs, dimerize with ARNT and activate HRE-dependent transcription. Hypoxia induces ECM remodelling through collagen modification (P4HA1/2, PLOD2), cross-linking (LOXL2), MMP activation and CA9-mediated acidification, promoting matrix stiffening and invasion. CAFs enhance immunosuppression via TGF-β–mediated myofibroblast transition and secretion of CXCL12, IL-6, IL-10, PD-L1 and ARG2, leading to T-cell exclusion and reduced activation. Metabolic reprogramming increases GLUT1-driven glycolysis, lactate export (MCT4), reduces α-KG, ROS signalling and HIF-1α stabilisation. Finally, CAF-derived VEGF, HGF and pro-inflammatory signalling promote angiogenesis, EMT, invasion and metastasis.

Thus, this complex regulatory network reprograms CAFs through hypoxia-induced transcriptional, epigenetic, metabolic and redox-dependent processes. HIF-1α plays a key role in linking oxygen sensing to ECM remodelling enzymes, glycolytic regulators, chemokines and angiogenic factors. These coordinated pathways enable hypoxic CAFs to shape a microenvironment marked by stiffness, acidosis, immune suppression, metabolic coupling, abnormal angiogenesis and increased metastatic potential. This detailed molecular framework is illustrated in Figure 1.

## HIF-driven metabolic reprogramming as a driver of immune suppression

**Figure 2.**
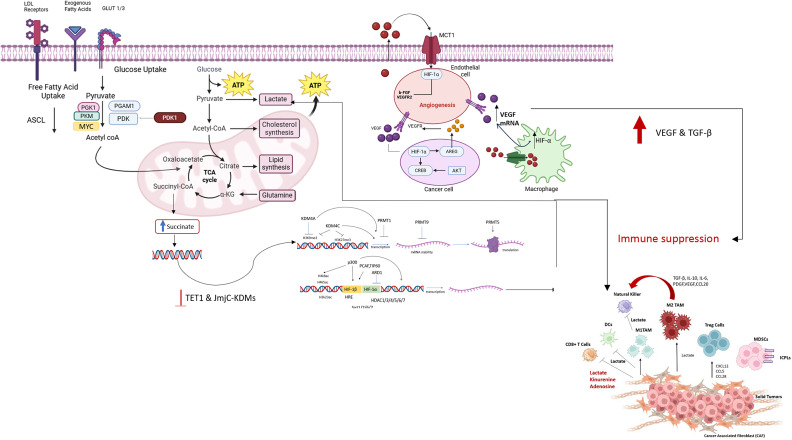
This schematic illustrates how hypoxia regulates angiogenesis, metabolic reprogramming and immune evasion in the tumour microenvironment (TME) through hypoxia-inducible factors HIF-1α and HIF-2α. Under low-oxygen conditions, HIF-1α activates endothelial cells (ECs) and promotes neovascular sprouting via vascular endothelial growth factor (VEGF) signalling, whereas loss of HIF-2α results in structurally abnormal, leaky blood vessels. Hypoxic tumour cells undergo metabolic reprogramming, characterised by a shift towards glycolysis, leading to increased lactate and succinate production. High levels of succinate expression lead to epigenetic plasticity, thereby increasing lactate. Lactate is transported out by monocarboxylate transporter 1 (MCT1) and taken up by ECs, enhancing their metabolism in a metabolic symbiosis that sustains angiogenesis. Significant signalling pathways such as HIF-1α/SNHG1/miR-199a-3p/TFAM and EPOR1/VEGFA also play roles in metabolic adaptation and tumour progression, particularly in breast cancer. At the same time, hypoxia enables immune evasion by prompting tumour cells and cancer-associated fibroblasts (CAFs) to release immunosuppressive metabolites – lactate, kynurenine and adenosine – as well as cytokines like VEGF, transforming growth factor-beta (TGF-β) and interleukin-10 (IL-10). These suppress the functions of cytotoxic CD8^+^ T cells, dendritic cells (DCs) and natural killer (NK) cells. The accumulation of lactate further skews macrophages towards an M2-like phenotype, supporting tumour growth and immune suppression. HIFs also enhance the differentiation of regulatory T cells (Tregs) and recruit myeloid-derived suppressor cells (MDSCs), intensifying immunosuppressive signals. Moreover, hypoxia increases the expression of immune checkpoint molecules such as programmed death-ligand 1 (PD-L1) and cytotoxic T-lymphocyte-associated protein 4 (CTLA-4), further inhibiting T cell function and diminishing the effectiveness of immunotherapies. Collectively, these processes illustrate the intricate interactions among metabolic, vascular and immune pathways in the hypoxic TME, highlighting potential therapeutic targets to disrupt tumour progression and resistance. CAF: cancer-associated fibroblast; CTLA-4: cytotoxic T-lymphocyte-associated protein 4; DC: dendritic cell; EC: endothelial cell; EPOR1: erythropoietin receptor 1; HIF: hypoxia-inducible factor; IL-10: interleukin-10; MCT1: monocarboxylate transporter 1; MDSC: myeloid-derived suppressor cell; miR-199a-3p: microRNA-199a-3p; NK cell: natural killer cell; PD-L1: programmed death-ligand 1; SNHG1: small nucleolar RNA host gene 1; TFAM: mitochondrial transcription factor A; TGF-β: transforming growth factor-beta; TME: tumour microenvironment; Treg: regulatory T cell; VEGF: vascular endothelial growth factor; VEGFA: vascular endothelial growth factor A.

Hypoxia-driven metabolic reprogramming is a central mechanism by which solid tumours within TME actively suppress anti-tumour immunity (Figure 2). To sustain rapid proliferation under low-oxygen conditions, tumour cells rewire glucose, lipid and amino acid metabolism, leading to nutrient depletion, lactate accumulation and increased H+ production, which acidifies the TME. These metabolic changes directly impair the function of T cells, NK cells and dendritic cells, thereby promoting immune evasion. This metabolic shift is commonly referred to as the Warburg effect (Ref. 53). The Warburg effect leads to the activation of MYC and HIF-1 in response to growth factors and hypoxia (Ref. 3). HIF-1α is the primary transcription factor promoting Warburg-like metabolism. It stimulates key enzymes involved in glycolysis, such as phosphoglycerate mutase 1 (PGAM1), pyruvate kinase M (PKM), phosphoglycerate kinase 1 (PGK1), LDHA, lactate dehydrogenase C and lactate dehydrogenase-5 (LDH-5), facilitating a shift to anaerobic metabolism (Ref. 54). Additionally, HIF-1α activates the mTOR signalling pathway, enhancing the expression of glucose transporters GLUT1 and GLUT3 to boost glucose uptake (Ref. 55). The activation of the mTOR /P13K signalling pathway was studied to induce secondary inhibition of T cell proliferation (Ref. 56). On the other hand, the immune resistance effect is further exacerbated under hypoxic conditions due to nutrient competition between tumour cells and immune effector cells, such as CD8+ and NK cells, which rely on glucose and glutamine for their anti-tumour functions, thereby suppressing effective immune responses. As hypoxia increases, tumour-associated macrophage (TAM) polarisation towards a tumour-permissive M2 phenotype determines the number of myeloid-derived suppressive cells (MDSC) infiltrating the tumour (Ref. 56) glucose uptake.

HIF-2α further enhances MYC target gene expression, including LDHA. Meanwhile, HIF-1α inactivates pyruvate dehydrogenase (PDH) by upregulating PDH kinase 1 (PDK1), which prevents pyruvate conversion to acetyl-CoA, thereby limiting TCA cycle entry and leading to lactate accumulation (Ref. 2). Inhibitors of PDK1, such as dichloroacetate (DCA) have been observed to reverse the glycolytic phenotype and sensitize the tumours to chemotherapy and radiotherapy (Ref. 57). This lactate buildup increases intracellular ATP levels and reduces hypoxia-induced reactive oxygen species (ROS), protecting cells from apoptosis. Additionally, the lactate produced in hypoxic TME has been implicated in the impaired differentiation of monocytes into dendritic cells (DC), in the loss of the cytolytic functions by natural killer (NK) cells, in the increase of M2 macrophages and MDSC that inhibit NK activity (Ref. 58). Lipid metabolism also becomes a key energy source when glucose is scarce. Under hypoxic conditions, HIF-1α upregulates fatty acid-binding proteins (FABP3, FABP4 and FABP7) to promote fatty acid uptake. Simultaneously, HIF-1α inhibits fatty acid β-oxidation by downregulating acyl-CoA dehydrogenases, restricting acetyl-CoA entry into the TCA cycle. However, hypoxia increases acetyl-CoA production by inducing acetyl-CoA synthase 2 (ACSS2), which converts acetate into acetyl-CoA. Since the TCA cycle entry is blocked, this acetyl-CoA is redirected towards lipid biosynthesis, including membrane phospholipids. Elevated ACSS2 expression is associated with greater tumour aggressiveness in breast cancer (Ref. 59). Additionally, lipid accumulation is understood to impede NK cell and CD8+ T cell anti-tumour activities. The accumulation of intracellular lipids via the lipid transporter CD36 promotes the pro-tumourigenic function of M2-like TAMs. Moreover, lactate/low pH alters the Ag-presenting ability of dendritic cells. This was further confirmed by the study conducted by Hussain et al., which showed that MDSCs and NK cells are compromised by the lactate/pH balance (Ref. 58). Elevated ACSS2/KATA2 expression has been a potential prognostic biomarker for breast cancer and brain metastasis, as it promotes histone acetylation, tumour growth and immune evasion (Refs. 60, 61).

In glucose- and lipid-deprived conditions, amino acids, especially glutamine, become vital energy sources. Glutaminolysis, the conversion of glutamine-derived glutamate into pyruvate, is common in the TME. The conversion of glutamine to glutamate orchestrates the function of CD4+ and CD8+ T cells against tumours. However, under hypoxia, c-Myc and HIF-2α upregulate glutaminase 1 (GLS1), enhancing TCA cycle activity and anabolic pathways that provide precursors for nucleotides, proteins and membrane components, as well as glutathione for redox balance (Ref. 62). Additionally, glutamine actively provides carbon and nitrogen sources that are crucial for cell proliferation and metastasis. Additionally, it exacerbates DC-dependent anti-tumour immunity via the glutamine/SLC38A2/FLCN axis, which potently orchestrates cDC1-mediated CD8+ T cell accumulation, thereby promoting anti-tumour immunity (Ref. 63).

Glutamine supports lipogenesis through two mechanisms: (Ref. 1) α-ketoglutarate (αKG), derived from glutamine, enters the TCA cycle and is converted to malate, which is transformed into pyruvate by malic enzyme, generating NADPH for lipid synthesis; and (Ref. 2) αKG is carboxylated to isocitrate by isocitrate dehydrogenase (IDH) (Ref. 64). mTOR inhibitors, such as rapamycin and its analogues, inhibit hyperactive HIF-mTOR signalling (Ref. 65). The cytosolic isoform, IDH1, reversibly converts αKG into isocitrate, promoting reductive glutamine metabolism, while mitochondrial IDH2 catalyses the oxidative conversion of isocitrate to αKG (Ref. 20). Hypoxia enhances IDH1 activity and suppresses IDH2, increasing isocitrate and NADPH production, which feed into citrate and acetyl-CoA synthesis for lipid biosynthesis (Ref. 10). Increased IDH2 expression promotes T cell deletion and improves CAR-T cell function, associated with altered epigenetic function. Furthermore, the glutamine/glutamate imbalance within the TME impedes M1 and promotes M2 macrophage function. The anti-tumour immunity of arginine metabolism is suppressed by intratumoural DCs and TAMs, which express arginase 1 (ARG1) that catabolizes arginine, leading to arginine depletion and immune suppression (Ref. 66). Mutations in IDH1/2 are clinically significant in gliomas and leukaemias, and IDH inhibitors, such as ivosidenib and vorasidenib, are FDA-approved (Ref. 67). NADPH also maintains redox homeostasis. Additionally, both HIF-1α and HIF-2α upregulate amino acid transporters such as xCT (SLC7A11) and LAT1 (SLC7A5 (Refs. 54, 68). Many tumours show increased LAT1 expression in response to hypoxia and HIF-2α overexpression. LAT1 and xCT are potential drug targets due to their unique upregulation in solid tumours; LAT1 inhibitors (JPH203) are currently under clinical trials (Ref. 69). HIF-2α activation also stimulates mTOR signalling, further enhancing glucose uptake via GLUT1, GLUT3 and GLUT4 (Refs. 70, 71). The regulation of metabolic reprogramming in cancer cells is crucial for cell survival and proliferation. Studies have shown that USP25, a deubiquitylating enzyme, is a critical regulator of pancreatic ductal adenocarcinoma (PDAC) growth and survival, essential for tumour maintenance. Inhibiting USP25 – either genetically or with drugs – significantly impaired PDAC growth while sparing normal pancreatic cells, and led to strong tumour regression in xenograft models. Mechanistically, USP25 stabilizes HIF-1α, a key transcription factor that promotes glycolysis and survival under hypoxic conditions, which are common in PDAC. When USP25 is depleted, HIF-1α activity and glycolysis are suppressed, leading to cancer cell death in the tumour’s hypoxic core. These findings reveal the USP25/HIF-1α axis as a crucial survival mechanism (Ref. 72). Additionally, studies show that hypoxia-induced metabolic reprogramming in the TME alters regulatory T cell (Treg) function, enabling them to use alternative energy sources and enhance their survival, proliferation and immunosuppressive activity. This promotes immune evasion and supports tumour progression (Ref. 73). Therapeutic strategies that modulate Treg metabolism or reprogram TAMs, such as CSF1R inhibitors, are being extensively studied to enhance immunotherapy efficacy in solid tumours (Ref. 74). Furthermore, TAMs develop an immunosuppressive, pro-tumour phenotype. Due to tumour-driven metabolic changes, TAMs adapt their metabolism – altering glucose, lipid and amino acid pathways – which enhances their ability to support tumour growth and suppress anti-tumour immunity. These metabolic shifts include increased glycolysis, fatty acid oxidation and amino acid metabolism (e.g., arginine, tryptophan, glutamine) (Ref. 75).

## HIF-induced angiogenesis

Tumour cells thrive in low-oxygen environments by activating survival mechanisms, the most critical of which is angiogenesis, the formation of new blood vessels. Hypoxia-induced metabolic reprogramming actively fuels angiogenesis by increasing glycolytic flux and lactate production, thereby stabilising hypoxia-inducible factors (HIFs) and enhancing proangiogenic signalling. This coordinated process ensures a continuous supply of oxygen and nutrients while facilitating tumour growth and metastasis (Ref. 7). At the molecular level, HIFs play a central role in driving angiogenesis. Under hypoxic conditions, HIFs activate the expression of pro-angiogenic factors and their receptors, including vascular endothelial growth factor (VEGF), VEGF receptors (VEGFR-1, VEGFR-2), basic fibroblast growth factor (bFGF), platelet-derived growth factor (PDGF), insulin-like growth factor 2 (IGF2) and epidermal growth factor (EGF) (Ref. 76). Metabolic by-products such as lactate act as signalling metabolites that enhance VEGF expression and endothelial cell migration, while extracellular acidification promotes vessel sprouting and remodelling. Importantly, lactate accumulation within the TME stabilizes HIF-1α via the inhibition of proplyl hydroxylases and sensitizes TAMs towards an M2 phenotype. This results in the suppression of CD8 + T cell and NK cell effector function through acidification-mediated impairment of cytokine production and cytotoxicity. Together, these factors orchestrate the formation of new blood vessels, providing a molecular link between hypoxia, metabolic reprogramming and immune suppression. (Ref. 11). VEGF is an immunosuppressive, as it can inhibit the function of T cells and increase the recruitment of regulatory T cells (Tregs) and MDSCs. It further hinders the differentiation and activation of dendritic cells (DC) (Ref. 76). Mechanistically, VEGF signalling through VEGFR2 on endothelial cells downregulates adhesion molecules such as ICAM-1 and VCAM-1, inducing a state of endothelial anergy that prevents efficient lymphocyte adhesion and trans endothelial migration. Additionally, VEGF interferes with NF-κB signalling in DC precursors, blocking their maturation and impairing their antigen-presenting capacity. Ziogas et al. observed that VEGF significantly reduced the cytotoxic activity of T cells derived from blood samples (Ref. 77). Furthermore, Voron et al. demonstrated that VEGF directly induced PD-1 expression on CD8 + T cells via the VEGFR2-PLCϒ-calciineurin-NFAT signalling pathway, promoting T cell exhaustion characterised by enhanced PD-1, CTLA-4, TIM-3 and LAG-3 expression (Ref. 78). Clinically, targeting the VEGF pathway has become the cornerstone of antiangiogenic therapy in solid tumours. Bevacizumab, a monoclonal antibody against VEGF, is an FDA-approved drug; however, resistance often arises via a compensatory mechanism involving pro-angiogenic factors like PDGF and FGF, a process sustained by persistent hypoxia and metabolic rewiring within the TME. Bevacizumab has the potential to increase B and T cell counts in patients with metastatic non-small cell lung cancer (NSCLC) (Ref. 79). Additionally, Sunitinib reduced the expression of IL-10, Foxp3, PD-1, CTLA4 and BRAF. This modified the TME, leading to shifts in cytokine and co-stimulatory molecule expression profiles that favoured T cell activation and Th1 responses. (Ref. 78). The deletion HIF-1α gene in endothelial cells (EC) has been reported to reduce neovascularisation and tumour growth by disrupting the VEGF-mediated autocrine loop in EC. Conversely, deletion of HIF-2α results in disorganised vasculature and poor perfusion. (Ref. 80). Beyond angiogenesis, HF-1α directly induces PD-L1 transcription through binding to hypoxia response elements in the PD-L1 promoter, suppressing CD8 + T cell activity, whereas HIF-2α contributes to immunoregulation by modulating cytokine production and maintaining vascular integrity. (Refs. 81, 82). The immunosuppressive molecules, such as TGFβ and VEGF induced due to hypoxia and low pH, lower the expression of adhesion molecules, thereby interfering with the ability of immune cells to adhere to and migrate across vessel walls to solid tumours (Ref. 83). Furthermore, tumour endothelium can selectively express Fas ligand (FasL) under VEGF andPGE2 stimulation, inducing angiogenesis of infiltrating CD8 + T cells while sparing regulatory T cells, thereby establishing selective immune exhaustion within the tumour vasculature. Hence, anti-VEGFR2 antibody enhanced the delivery of immune cells to tumours, reprogrammed the TAMs from pro-tumour to anti-tumour phenotype and improved the vaccine therapy in murine breast cancer models (Ref. 84). HIF-1α and HIF-2α are being studied for selective inhibitor strategies in solid tumours, for instance, the HIF-2α-specific inhibitor belzutifan (MK-6482) and DFF332 (Refs. 85, 86). Studies have identified several signalling pathways activated by hypoxia in solid tumours that promote angiogenesis and metastasis. The regulatory axis involving HIF-1, SNHG1, miR-199a-3p and TFAM plays a significant role in tumour angiogenesis and metastasis in breast cancer (BC) under hypoxic conditions. Measurement of SNHG1 expression in human BC cells exposed to hypoxia (1% O2 for 24 hours) revealed elevated levels in a HIF-1-dependent manner. Knocking down SNHG1 resulted in decreased proliferation, migration, invasion, angiogenesis and lung metastasis in MDA-MB-231 cells. SNHG1 co-expressed with miR-199a-3p and regulated TFAM, which is a target gene of miR-199a-3p, by binding and increasing TFAM levels. These results highlight a regulatory axis of HIF-1, SNHG1, miR-199a-3p and TFAM in breast cancer development and metastasis under hypoxic conditions (Ref. 87). Additionally, hypoxia promoted angiogenesis and bone regeneration through the HIF-1/β-catenin pathway in bone marrow stem cells. Moreover, the loss of EPOR1, a protein disulfide that promotes proliferation in breast cancer via VEGFA, is expressed drastically in breast cancer under hypoxic conditions, thereby enhancing angiogenesis and proliferation (Ref. 88). Collectively, hypoxia-driven metabolic reprogramming promotes aberrant angiogenesis, not only supporting tumour growth but also establishing an immunosuppressive microenvironment. Persistent hypoxia sustains HIF-activation, drives VEGF-mediated endothelial dysfunction, enforces metabolic competition through lactate accumulation, induces immune checkpoint expression and restricts cytotoxic immune trafficking, thereby creating a self-reinforcing feedback loop between angiogenesis and immune evasion within the tumour niche.

## Immunometabolic mechanism of HIF-mediated immune resistance

Hypoxia enables malignant tumours to evade immune surveillance by both suppressing the activity of anti-tumour immune cells and promoting immunosuppressive elements within the TME. Metabolic reprogramming within the TME shifts nutrient availability and metabolite composition, thereby directly constraining immune cell survival and effector function. Mechanistically, HIF-mediated immune resistance operates through four coordinated axes: (1) intrinsic suppression of effector immune cell metabolism and function, (2) recruitment and stabilisation of immunosuppressive cell populations, (3) induction of immune checkpoint signalling and (4) structural and vascular remodelling that restricts immune cell trafficking. HIF signalling, therefore, integrates metabolic stress with transcriptional programmes that promote immune escape. (Ref. 6).

HIF-1α impairs the survival and functionality of dendritic cells (DCs), natural killer (NK) cells and cytotoxic CD8^+^ T cells. The primary function of dendritic cells is to present tumour-derived antigens to naïve T cells to initiate adaptive immune responses. Hypoxia limits the ability of mature Dcs to capture antigens by downregulating Rho GTPases and ERM proteins (Ref. 89). The Warburg effect is modulated by the RhoA/Rho-associated protein kinase signalling axis. Additionally, Rho GTPases activate mitochondrial glutaminases (GLS1), an enzyme that catalyses the first step of glutaminolysis. Thereby affecting the adaptive immune response due to hypoxia (Refs. 90, 91).

MDSCs represent a key immunosuppressive population in the TME. Under hypoxic conditions, splenic MDSCs exhibit selective and rapid upregulation of PD-L1 via direct binding of HIF-1α to an HRE in the PD-L1 promoter. Chromatin immunoprecipitation and luciferase reporter assays confirm this regulatory mechanism. HIIF-1α serves as a central metabolic regulator in MDScs, linking immune checkpoint expression to hypoxia-driven metabolic reprogramming. Under low oxygen tension. Here, there is a shift towards aerobic glycolysis via upregulation of glycolytic enzymes, such as GLUT1, HK2 and LDHA, leading to increased lactate production (Ref. 92). Blocking PD-L1 under hypoxia restores T cell activation and suppresses IL-6 and IL-10 production by MDSCs. Furthermore, neutralisation of IL-10 abrogates MDSC-mediated suppression, reinforcing the role of hypoxia in regulating both immune checkpoints and cytokine-mediated suppression (Refs. 24, 93).

In vivo studies have shown that inhibiting HIF-1α transcriptional activity or disrupting the HIF-1α/HIF-1β dimer significantly impedes melanoma growth (B16-F10 model), enhances infiltration of CD8^+^ T cells and NK cells and promotes chemokine release (CCL2 and CCL5), thereby boosting anti-tumour immunity (Ref. 24). In CD4^+^ T cells, HIF-1α directly binds the FOXP3 promoter, driving regulatory T cell (Treg) differentiation via TGF-β-dependent pathways. TGF-β activates SMAD2/3, which cooperates with HIF-1α at the FOXP3 locus to enhance transcriptional activity and promote regulatory T cell differentiation. Concurrently, hypoxia induces a metabolic shift towards glycolysis by upregulating GLUT1, hexokinase 2 and lactate dehydrogenase A (LDHA). Although Tregs rely on oxidative phosphorylation and fatty acid oxidation (FAO) for long-term stability, hypoxia-driven glycolytic intermediates support early Treg differentiation, while enhanced fatty acid uptake via CD36 and FAO sustains their suppressive phenotype. This Treg skewing is metabolically reinforced by hypoxia-induced glycolysis and fatty acid utilisation, which supports Treg survival and limits T cell metabolism. This mechanism not only enhances immune tolerance but also contributes to EMT and tumour proliferation by activating SMAD3 (Refs. 94, 95).

The hypoxic TME also disrupts immune responses metabolically and structurally. It fosters an acidic environment through increased glycolysis and lactate accumulation. HIF-1α upregulates carbonic anhydrases (CAIX, CAXII), Na^+^/H^+^ exchangers and bicarbonate transporters, maintaining acidosis that impairs T cell and NK cell cytotoxicity by inhibiting NFAT activation (Ref. 96). Lactate accumulation hampers DC maturation, shifts macrophages towards an M2 phenotype and inhibits T cell proliferation and effector function (Ref. 97). This tumour-infiltrating DC phenotype in the TME is influenced by tumour-derived lactate. The antigen MHC-1 complex on DCs is more suitable for a neutral environment; therefore, the acidified TME hinders the antigen uptake capacity of DCs and stability of the antigen MHC-1 Complex. Therefore, high lactate levels interfere with T cell metabolism and antitumour immune function. Indicating that lactate, a metabolic byproduct of aerobic glycolysis in cancer cells, can induce apoptosis and primary T cell downregulation (Ref. 98). Additionally, CD8^+^ T cells struggle to export lactate under hypoxic conditions, leading to intracellular acidification and reduced function (Ref. 99).

Nutrient competition represents a critical metabolic checkpoint of immune suppression. Hypoxia in tumour cells upregulates glucose transporters (GLUT1) and glutaminase via HIFα and c-Myc signalling, outcompeting immune cells for glucose and glutamine. (Refs. 100, 101). As a result, immune cell metabolism is compromised, leading to anergy or apoptosis.

Immune checkpoint proteins (ICPs) are tightly regulated under hypoxia. HIF-1α and HIF-2α directly upregulate PD-L1 on tumour and stromal cells and enhance expression of PD-1, CTLA-4 and LAG-3 on T cells (Ref. 102). PD-L1 expression is also driven by Akt/mTOR signalling. Blocking PD-1/PD-L1 restores glucose uptake in T cells, reversing exhaustion (Ref. 91). PD-1 signalling promotes fatty acid oxidation, a less efficient metabolic state for T cell effector function, reversible with checkpoint blockade (Ref. 103).

Post-translational mechanisms also support immune evasion. Hypoxia alters PD-L1 glycosylation and enhances its palmitoylation, stabilising its membrane localisation. Proteins such as CMTM6 and ARF6 facilitate PD-L1 trafficking, while alterations in membrane lipids affect checkpoint receptor–ligand interactions (Ref. 104). Recent studies have identified palmitoylation inhibitors and glycosylation modulators as potential targets for ICP therapy, as they destabilize PDL-1 and enhance ICPI efficacy (Ref. 105). These changes are especially relevant in cancers like clear cell renal cell carcinoma (ccRCC), where HIF-2α, in the context of VHL loss, acts as a major transcriptional driver of PD-L1, alongside STAT3 activation and PTEN loss (Ref. 106). Belzutifan, a selective inhibitor of HIF-2α, has been approved by FD since 2021, and currently, as of 2023 it is under investigation for the combined use with ICIs for advanced ccRCC, showing promising synergistic effects in reducing PDL-1 driven immune evasion (Ref. 107).

Hypoxia also fosters a pro-angiogenic, immunosuppressive loop via HIF-1α-driven expression of VEGF, PDGF-β, ANGPT2, PGF and CXCL12. These factors support tumour neovascularisation and recruit suppressive immune populations, including Tregs, MDSCs and TAMs. VEGF additionally impairs DC maturation, promotes PD-L1^+^ TAMs and increases PGE2 production via COX2, suppressing NK cells and enhancing immune evasion (Ref. 108). Dual inhibition strategies targeting VEGF and COX2/PGE2 signalling are being studied for their antitumour properties by inhibiting PDL-1 and sensitising tumours to immune checkpoint blockade (Ref. 109).

Collectively, these mechanisms severely limit the efficacy of immune checkpoint inhibitors (ICPIs). Clinical and preclinical studies demonstrate that tumours in oxygenated regions respond better to ICPIs, independent of PD-L1 expression or T cell infiltration (Ref. 104). In gliomas and melanomas, ICPI efficacy is markedly enhanced in normoxic environments, reinforcing hypoxia as a barrier to immunotherapy. Targeting hypoxia pathways, such as HIF-1α, metabolic reprogramming or chemokine signalling holds promise for overcoming ICPI resistance and restoring anti-tumour immunity (Ref. 102).

## Epigenetic reinforcement of HIF-driven metabolic and immune adaptations

The transcriptional activity of HIFs is controlled by various epigenetic regulators, such as, TET1, ALKBH5, Jmjc-demethylases, HDAC3, WDR5, G9a, GLP, SIRT1–7 and KDM5B,3A,4A and 6B. The stability of HIF-α subunits underpins the biological functions of HIF-α complexes, which, in turn, regulate hypoxia-related phenotypes in cancer cells. Therefore, epigenetic regulation of HIF is often described as a form of hypoxic memory in the TME that sustains metabolic rewiring and immune evasion over time. (Ref. 110).

DNA and histone methylation are crucial epigenetic regulators in hypoxic tumours. As it influences the stability of HIF, thereby regulating the of HIF target genes, such as the silencing of the tumour suppressor gene. For instance, hypermethylation of the VHL promoter enhances transcriptional activation of HIF-1α and promotes HIF-1α target gene activation, such as carbonic anhydrase 9 (CA9) and glucose transporter type 1 (GLUT1) (Ref. 111). Moreover, TET1 enhances polyubiquitination of HIF-α by altering lysine site modifications and stabilising HIF-1α, thereby activating HIF signalling, which enhances EMT transition and angiogenesis in neuroblastoma cells (Ref. 26). Histone methyltransferase, G9a and GLP, catalyse H3K9 methylation, resulting in increased proliferative and invasive potential of breast cancer cells (Ref. 112). H3K9 methylation reinforces glycolytic gene expression and suppresses mitochondrial oxidative metabolism, favouring a Warburg-like metabolic phenotype. This chromatin state also silences antigen presentation machinery, contributing to enhanced immune evasion. Furthermore, protein arginine methyltransferase 2 (PRMT2) is a DNA methylation transcription factor that is induced during chronic hypoxia. Mice exposed to hypoxia showed increased mRNA and protein levels of PRMT2. PRMT2 supports hypoxia adaptive transcriptional programmes linked to metabolic stress tolerance (Ref. 113). Furthermore, KDM3A is reported to enhance glycolysis by interacting with HIF-1α to modulate phosphoglycerate kinase 1 (PGK1) transcription, thereby reducing H3K9me2 levels at the PGK1 HRE promoter. Elevated glycolytic flux mediated by KDM3A further exacerbates nutrient competition, depriving immune cells of glucose required for effector function (Ref. 114).

Histone acetylation is a common marker of gene activation, whereas histone deacetylation is a marker of repression. Histone acetyltransferases (HATs) induce acetylation of the amino group of lysine residues in histone tails. Histone acetylation, carried out by HATs, loosens DNA-histone interactions to promote gene activation, while histone deacetylases (HDACs) reverse this process to repress transcription. HATs are grouped into five families – GNAT, p300/CBP, HAT1, Rtt109 and MYST – based on structure and function. These enzymes target specific lysine residues, such as H3K9, H3K14 and H4K12. GNAT members (e.g., GCN5, PCAF) and p300/CBP proteins have bromodomains for protein binding, while MYST family enzymes (e.g., TIP60, MOZ) contain chromodomains for interaction. In contrast, HDACs are categorised into four classes (Ref. 115). Classes I, II and IV use zinc as a cofactor, whereas class III (sirtuins) rely on NAD^+^. Class I HDACs (HDAC1–3, 8) mainly act in the nucleus, often as part of large complexes, while Class II HDACs (e.g., HDAC4–10) have specialised domains for localisation and interaction. Class III HDACs (SIRT1–7) have diverse roles across cellular compartments, and HDAC11 is the sole, less-studied member of Class IV (Ref. 116). However, under hypoxia, HATs affect HIF-1α stability and hypoxia-induced transcriptional activity. P300/CBP interacts with HIF-1α to recruit to the promoters of EPO and VEGF, enhancing Gene activation under hypoxia and thus increasing cell proliferation. Beyond the metabolic and angiogenic reprogramming, p300/CBP-mediated acetylation of HIIF-1α has been implicated in immune suppression by enhancing the transcription of immune-evasive genes, such as PDL-1, and impairing cytotoxic T cell recognition (Ref. 117). Moreover, HAT1, a type B acetyltransferase, is an essential gene in glioblastoma under hypoxia, as it stabilizes the HAT1-HIF2α axis that promotes cancer stemness and reprogramming. Furthermore, it indirectly contributes to immune suppression by downregulating the MHC expression, thereby inducing resistance to immune-mediated clearance. (Ref. 118). Histone deacetylases interact with the ODD domain of the HIF-1α, increasing the expression of HIF-1α under hypoxia. HDAC1 and HIF-1α form a complex that binds to the promoter regions of certain long non-coding RNAs (lncRNAs), leading to histone H3 deacetylation and suppression of lncRNA expression. One such lncRNA, CF129, is repressed by this complex, which promotes p53 degradation. The resulting accumulation of p53 enhances the expression of FOXC2 and HIF-1α (Ref. 119). Additionally, hypoxia decreases the expression of another lncRNA, FAM99A, which normally suppresses EMT in hepatocellular carcinoma by targeting miR-92a. This downregulation is also driven by HDAC1 and HIF-1α, leading to increased deacetylation at the FAM99A promoter. Together, these findings suggest that HDAC1 contributes significantly to hypoxia-induced metastasis via the HIF-1α/HDAC1/FAM99A/miR-92a/E-cadherin regulatory pathway. EMT induction under hypoxia is directly linked to immune suppression, as it is characterised by decreased T cells, increased secretion of immunosuppressive cytokines, and resistance to immune checkpoint inhibitors (Refs. 22, 120). Furthermore, SIRT3 interacts with HIF-1α and the SRT3/K741 axis, and it enhances cell survival and angiogenesis in endothelial cells under hypoxia (Ref. 121). HIF-1 α drives immune escape in triple-negative breast cancer by inducing HIF1α-mediated epigenetic suppression of immune effector genes. Hypoxia impairs T and NK cell function by enabling HIF1α to interact with HDAC1 and PRC2, leading to chromatin remodelling and silencing of key immune genes. Therefore, this coordinated histone deacetylation-dependent repression of immune stimulatory pathways leads to dysfunctional anti-tumour immunity and promotes resistance to immunotherapy in hypoxic tumours (Ref. 4).

Taken together, these findings underscore hypoxia as a central driver of immune resistance through coordinated metabolic, angiogenic and epigenetic reprogramming. The growing understanding of HIF-mediated pathways has prompted the development of therapeutic strategies to disrupt hypoxia signalling and restore anti-tumour immunity. Importantly, combinatorial approaches integrating hypoxia-targeted agents with immune checkpoint inhibitors and metabolic modulators are emerging as promising strategies to overcome resistance and enhance therapeutic efficacy. Representative clinical and preclinical approaches targeting these interconnected pathways are summarised in Table 1.

**Table 1.** Combinatorial hypoxia-targeted therapies with immune checkpoint and metabolic modulation strategies

Hypoxia-targeted therapy (target)	+ Immunotherapy	+ Metabolic inhibitors		References
HIF–2α inhibitor: belzutifan (MK–6482/WELIREG)	Belzutifan + pembrolizumab (adjuvant clear cell renal cell carcinoma)	N/A	Renal cell carcinoma	NCT03401788 (Ref. 122)
Adenosine axis (hypoxia → adenosine accumulation): A2A receptor antagonists (ciforadenant/CPI–444)	Ciforadenant + atezolizumab (PD-L1)	N/A	Renal cell carcinoma	(Ref. 123) NCT02655822
Adenosine axis: A2A receptor antagonist AZD4635	AZD4635 + durvalumab (PD-L1) ± oleclumab (CD73)	N/A	Non-Small Cell Lung Cancer and Colorectal Cancer	NCT04089553 (Ref. 124)
CD73 inhibition (ecto–5′-nucleotidase): oleclumab (MEDI9447)	Durvalumab (PD-L1) + oleclumab	N/A	Non-Small Cell Lung Cancer	(Ref. 125) NCT04262388
CD73 inhibition: CPI–006 (anti-CD73 monoclonal antibody)	CPI–006 + pembrolizumab (PD–1); also evaluated with A2A antagonists	N/A	Advanced solid tumours	NCT03454451 (Ref. 126)
CD39 inhibition (upstream of adenosine production): TTX–030	TTX–030 + pembrolizumab (PD–1) (Phase 1/1b)	N/A	Thyroid cancer	NCT04306900 (Ref. 127)
CD39 inhibition: IPH5201 (anti-CD39 monoclonal antibody)	IPH5201 + durvalumab (PD-L1)	N/A	Non-Small Cell Lung Cancer	NCT05742607
GLUT1 inhibition (hypoxia-driven glycolysis): BAY–876, WBZ117	N/A	GLUT1 inhibitors + HIF–1 inhibitors in pancreatic solid tumours (preclinical Synergy)	Colorectal cancer	(Ref. 125)
Carbonic anhydrase IX (CAIX) inhibition: SLC–0111	N/A	SLC–0111 + GPX4 inhibitor (RSL3) (preclinical metabolic-redox targeting)	Pancreatic ductal cancer	NCT03450018
Acidosis/hypoxia adaptation: carbonic anhydrase inhibition (acetazolamide)	OBX–115 (engineered TIL therapy) + acetazolamide	N/A	Metastatic melanoma	NCT05470283
HIF–1α inhibition: PX–478	PX–478 + anti-PD–1 (preclinical synergy)	N/A	Non-Small Cell Lung Cancer	(Ref. 128)

## Conclusion

Hypoxia plays a key role in tumour progression, altering the TME through interconnected mechanisms of immune suppression, metabolic reprogramming, abnormal angiogenesis and epigenetic changes – mainly driven by HIFs. HIF-1α functions as a central system-level regulator that coordinates transcriptional, metabolic and immune adaptations, thereby enabling tumour cells to survive and expand in low-oxygen environments. Reduced oxygen availability within the TME drives profound metabolic rewiring, characterised by enhanced glycolysis, lactate build-up and exacerbated competition for nutrients. Thereby fostering a hostile, acidic environment that is required for rapid tumour proliferation and immune suppression. Thereby resulting in impaired cytotoxic T-cell and NK cell function, immune checkpoint molecules such as PD-L1 are upregulated, and encourage the recruitment of immunosuppressive cells, including regulatory T cells (Tregs), MDSCs and tumour-associated macrophages, creating a niche resistant to immune responses. On the other hand, HIF-induced angiogenesis results in abnormal blood vessels that restrict immune cell infiltration and enhance metastasis. Beyond these immediate adaptive responses, hypoxia induces epigenetic reprogramming, such as histone modifications, and disrupts non-coding RNAs, stabilising gene expression programmes that facilitate immune evasion and resistance to therapy. These epigenetic alterations act as a molecular memory of hypoxic stress, allowing tumour cells to ‘remember’ and maintain hypoxia-adapted immunosuppressive states. As a result, metabolic rewiring, immune suppression and epigenetic memory form a reinforcing adaptive loop under hypoxia, thereby driving the aggressive nature of the tumour and inducing immunotherapy resistance. Therefore, future research should focus on rational drug combinations in hypoxic tumours and establish robust hypoxia-based biomarkers to guide precision medicine. Targeting hypoxia-driven immune suppression and metabolic dysfunction is essential for converting the TME into an immunoreactive environment and achieving lasting responses in therapy-resistant cancers.
